# OA03 An unusual cause of fragility neck of femur fracture in a 24 years old young adult: severe Vitamin D deficiency and Osteomalacia.

**DOI:** 10.1093/rap/rkag113.003

**Published:** 2026-11-03

**Authors:** Zina Afroja, Saima Almas, Chisom Nwanejuafor, James Taylor, Rachel Jeffery

**Affiliations:** Northampton General Hospital, Northampton, United Kingdom; Northampton General Hospital, Northampton, United Kingdom; Northampton General Hospital, Northampton, United Kingdom; Northampton General Hospital, Northampton, United Kingdom; Northampton General Hospital, Northampton, United Kingdom

## Abstract

**Introduction:**

Young adult neck of femur fractures following low-energy trauma are rare and should prompt urgent investigation for underlying metabolic bone disease or secondary osteoporosis. Although vitamin D deficiency is one of the most common reversible causes of impaired bone mineralisation, severe deficiency may remain clinically silent until presentation with a fragility fracture. We present the case of a 24-year-old man with a low-energy intracapsular neck of femur fracture secondary to severe vitamin D deficiency and nutritional osteomalacia

**Case description:**

A 24-year-old previously healthy man presented to the Emergency Department on 2 February 2025 after a low-impact fall at home. He slipped on a wet kitchen floor, landing on his left hip, and immediately developed severe pain with inability to weight bear. Radiographs demonstrated a displaced intracapsular fracture of the left neck of femur, and he underwent urgent dynamic hip screw fixation with an uncomplicated recovery.

The low-energy mechanism prompted investigation for underlying metabolic bone disease. His medical history included orthognathic surgery in 2022 and fractures of the third and fourth metacarpals following a fall at age 18. There was no family history of fractures or inherited bone disease. He worked as a restaurant manager and reported a calcium- and vitamin D-poor diet, avoidance of dairy products, limited sunlight exposure and smoking three cigarettes daily. He did not consume alcohol. Although he recalled being smaller than his peers during childhood, examination revealed no features suggestive of inherited skeletal or connective tissue disorders.

Laboratory investigations demonstrated profound vitamin D deficiency (25-hydroxyvitamin D < 20 nmol/L), hypocalcaemia (corrected calcium 2.16 mmol/L) and hypophosphataemia (0.78 mmol/L), consistent with osteomalacia. Despite these abnormalities, he denied preceding bone pain, proximal muscle weakness or functional limitation.

High-dose cholecalciferol 50,000 IU weekly was commenced, followed by maintenance cholecalciferol (800–1,000 IU daily) with calcium and vitamin D supplementation. Serum calcium and phosphate normalised after treatment. Dual-energy X-ray absorptiometry showed reduced bone mineral density with lumbar spine T-score −2.9, total hip T-score −1.4). Given his young age and the presence of a reversible metabolic cause, anti-osteoporotic therapy was not initiated. Ongoing management focused on correcting vitamin D deficiency, lifestyle modification and serial biochemical and DXA monitoring to assess skeletal recovery.

**Discussion:**

Low-energy femoral neck fractures in young adults are uncommon and should always prompt investigation for underlying metabolic bone disease. Although vitamin D deficiency is frequently encountered in clinical practice, presentation with a fragility neck of femur fracture in an otherwise healthy 24-year-old is exceptionally rare.

Vitamin D deficiency results in impaired intestinal calcium absorption, secondary hyperparathyroidism, phosphate wasting and defective mineralisation of newly formed osteoid, ultimately leading to osteomalacia. Classical clinical features include proximal muscle weakness, diffuse bone pain, fatigue, and pseudofractures, however, this case demonstrates that profound deficiency may remain clinically silent until a major fragility fracture occurs. His limited sunlight exposure and smoking were important modifiable risk factors contributing to impaired bone health. Clinical assessment did not identify features suggestive of inherited skeletal disorders such as osteogenesis imperfecta or other causes of secondary osteoporosis. The combination of severe vitamin D deficiency, biochemical abnormalities, radiographic osteopenia and reduced bone mineral density on DXA strongly supported nutritional osteomalacia as the underlying diagnosis.

Management focused on correction of vitamin D deficiency and restoration of calcium homeostasis. In young adults with osteomalacia, correction of the underlying mineralisation defect is the priority. Anti-resorptive therapy is generally not indicated in the absence of primary osteoporosis, as bone density often improves following correction of vitamin D deficiency and calcium homeostasis. Long-term calcium and vitamin D supplementation together with serial biochemical and DXA monitoring were recommended to assess skeletal recovery.

This case emphasises the importance of considering osteomalacia in any young adult presenting with a fragility fracture, even in the absence of classical symptoms and highlights the need for a systematic evaluation for secondary osteoporosis before initiating long-term osteoporosis therapy.

**Key learning points:**

• Low-energy neck of femur fractures are rare in young adults and should always prompt urgent investigation for underlying metabolic bone disease or secondary osteoporosis rather than being attributed solely to the mechanism of injury. Severe vitamin D deficiency may remain clinically silent and present without the classical manifestations of osteomalacia, including proximal muscle weakness, diffuse bone pain or functional limitation, until a major fragility fracture occurs. Nutritional osteomalacia is an important and potentially reversible cause of fragility fractures in young adults, particularly in those with poor dietary calcium intake, limited sunlight exposure and modifiable lifestyle risk factors such as smoking. Young adults presenting with fragility fractures should undergo an appropriate assessment for other secondary causes of reduced bone mineral density, including inherited disorders (such as osteogenesis imperfecta), endocrine, renal, gastrointestinal and inflammatory conditions, where clinically indicated. Diagnosis is supported by characteristic biochemical abnormalities, including profoundly reduced 25-hydroxyvitamin D, hypocalcaemia, hypophosphataemia, together with reduced bone mineral density on DXA. When a reversible metabolic cause is identified, management should prioritise correction of the underlying abnormality with high-dose vitamin D replacement, adequate calcium supplementation, lifestyle modification (including smoking cessation and optimisation of dietary calcium intake and sunlight exposure) and longitudinal monitoring with serial biochemistry and DXA. Anti-resorptive therapy is generally not indicated in young adults when nutritional osteomalacia is the primary underlying diagnosis. Early recognition and treatment can improve bone mineral density, reduce future fracture risk and reinforce the importance of public health measures aimed at preventing vitamin D deficiency in at-risk populations.

